# A rare cause of massive gastrointestinal bleeding in a renal transplant recipient—CMV small-bowel ulcer: a case report

**DOI:** 10.3389/fmed.2026.1959416

**Published:** 2026-10-20

**Authors:** Tony Lam, Kwok Yung Yuen

**Affiliations:** 1 Department of Medicine, Yan Chai Hospital, Hospital Authority, Hong Kong SAR, China; 2 Department of Microbiology, The University of Hong Kong Li Ka Shing Faculty of Medicine, Hong Kong SAR, China

**Keywords:** case report, CMV, renal transplant, small-bowel bleeding, small-bowel ulcer

## Abstract

A 26-year-old renal transplant recipient presented to our unit with passage of fresh and altered blood. He was on multiple immunosuppressants including mycophenolate mofetil, tacrolimus, and prednisolone. He was profoundly anaemic on admission, requiring up to 10 units of packed cell transfusion. Oesophagogastroduodenoscopy and colonoscopy showed no bleeding source. Subsequent enteroscopy showed several ulcers in the ileum, while biopsy showed cytomegalovirus viral inclusions. He was treated with a 3-week course of valganciclovir. Re-scope 1 month after the enteroscopy showed that the ulcers were largely healed.

## Introduction

We report a case of cytomegalovirus (CMV) small-bowel ulcer causing massive gastrointestinal bleeding in a renal transplant recipient.

Immunosuppression, as in transplant recipients, is a known risk factor for CMV infection and reactivation. CMV disease can involve every organ or system. The gastrointestinal tract accounts for about 30% of all tissue-invasive CMV disease (1). It can involve any part, from the mouth to the anus (2). The colon and rectum are the most commonly involved parts of the gastrointestinal tract, followed by the stomach and oesophagus (3). The small bowel is rarely involved (3).

There are case reports of CMV involvement of the small bowel, presenting with bleeding (4), perforations (5), and obstructions (6). These reports often describe diffuse erythema with or without erosion, while discrete ulcers, as in our patient, are relatively rare.

## Case description

### Background

This patient had a known history of C1Q nephropathy with focal segmental glomerulosclerosis since childhood, and had received a renal transplant in 2021. He had been put on immunosuppressants since then. On admission, he was on mycophenolate mofetil (MMF) 180 mg twice a day, tacrolimus 10 mg daily, and prednisolone 5 mg daily. He also had thalassaemia minor, with a baseline haemoglobin (Hb) level of around 10–11 g/dL.

He presented with passage of fresh and altered blood, up to six times per day in the past 2 days. He had no abdominal pain, vomiting, or fever. He also had general malaise, compatible with anaemic symptoms. There was no suspicious food intake, and his travel history was negative.

He had never consumed alcohol. He denied the use of any over-the-counter drugs or herbs.

### Initial workup

On admission to the surgery unit, his haemoglobin level was 5.1 g/dL. A digital per-rectal exam showed altered blood, while proctoscopy showed bleeding from above, with no rectal or anal lesion visible. He was put on supportive transfusion. Urgent oesophagogastroduodenoscopy (OGD) was arranged on the first day of admission, which showed no bleeding. Computed tomography arteriography (CTA) was also performed on the same day, showing no contrast extravasation, with no identifiable lesion in the gastrointestinal tract. Colonoscopy (CLN) was performed the next day, showing pools of blood-stained fluid with clots along the whole length of the colon. Colonoscopy reached 20 cm proximal to the ileocaecal valve (ICV), showing trace blood-stained fluid from above; no mucosal lesion was seen.

### Further progress

The patient continued to have passage of blood 1–3 times per day after admission, and was given supportive transfusions. A total of 10 units of packed cells were given on the first 5 days of admission, and his Hb levels were maintained at around 7–8 g/dL.

Based on the OGD and CLN findings, small-bowel bleeding was suspected. The medical gastroenterology team was consulted, and capsule endoscopy was arranged for him on day 5 of admission. The capsule endoscopy showed that most of the ileum, and the whole length of the colon, was filled with altered blood, with a large amount of blood clots. Thus, small-bowel bleeding was suspected. He was transferred to the medical unit for further management.

At this juncture, the renal team was consulted, who suggested stopping MMF and continuing other immunosuppressants. This was because there is evidence of MMF being associated with small-bowel bleeding (7–9). Therefore, MMF was stopped on day 8 of admission. The serum tacrolimus level was checked and found to be within therapeutic range. Misoprostol was initiated on day 8 of admission; this was an off-label use, the rationale for which is outlined in later sections.

Clinically, the patient’s bleeding was gradually subsiding, with no more passage of blood as of day 9 to 10 of admission. His Hb levels were static and he did not require further blood product transfusions.

Double-balloon enteroscopy (DBE) via the anal route was arranged on day 12 of admission. We managed to reach 80 cm proximal to the ICV. Several ulcers, the largest being 1.5 cm in diameter, were seen in the distal ileum, with mildly adherent blood clots. These ulcers had regular margins, sloped edges and clean bases, with mild surrounding erythema (see Figure 1). There was no active bleeding. Multiple biopsies were taken from these ulcers.

**Figure 1:**
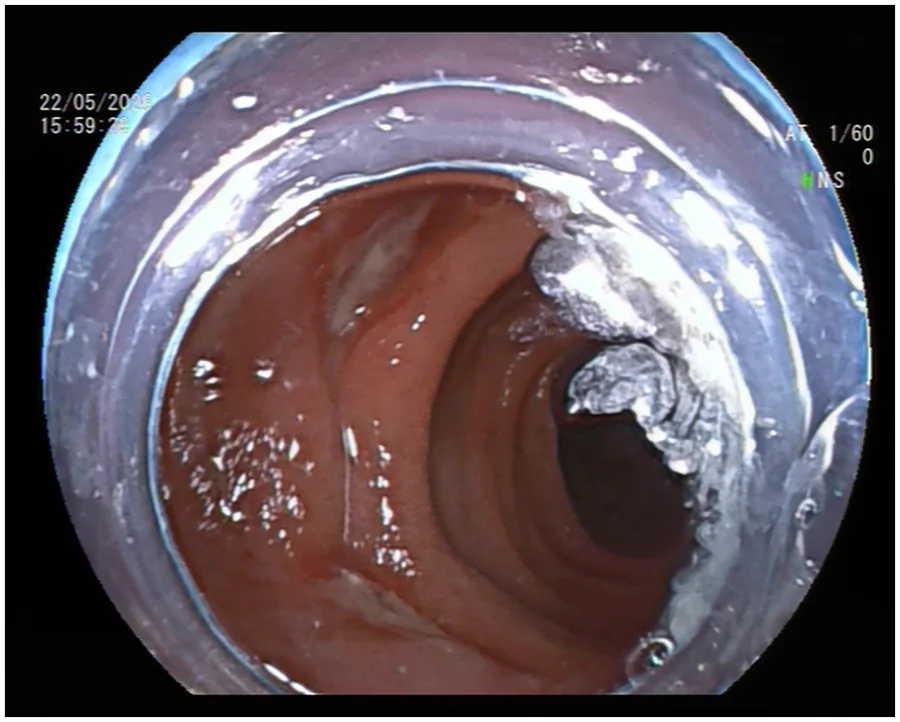
Endoscopic view of the small bowel ulcers.

Histology showed small intestinal mucosa with marked inflammation. CMV viral inclusions were seen (see Figure 2), and a CMV immunostain was positive (see Figure 3).

**Figure 2:**
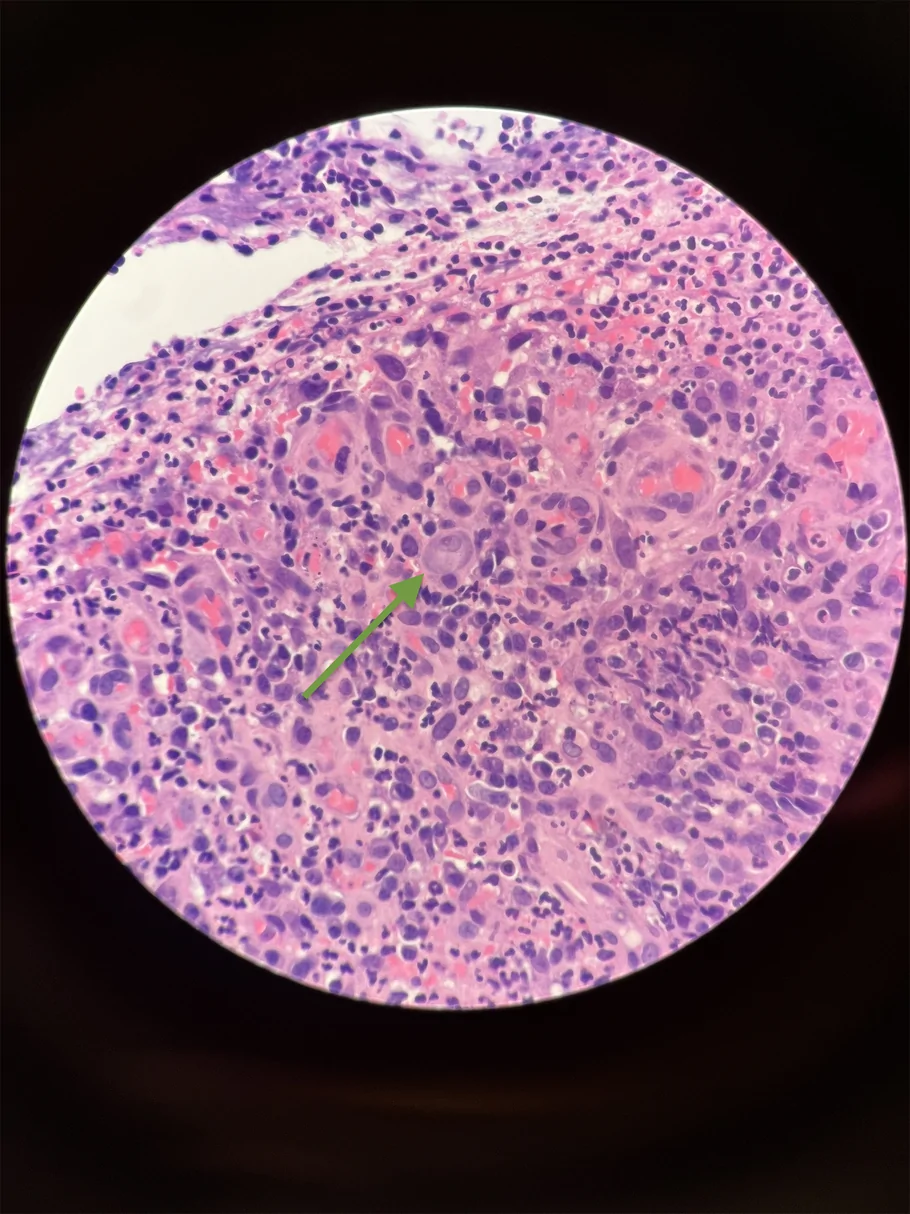
Histological image of the small bowel ulcer, showing CMV inclusion body (arrow), Hematoxylin and eosin (H&E) stain.

**Figure 3:**
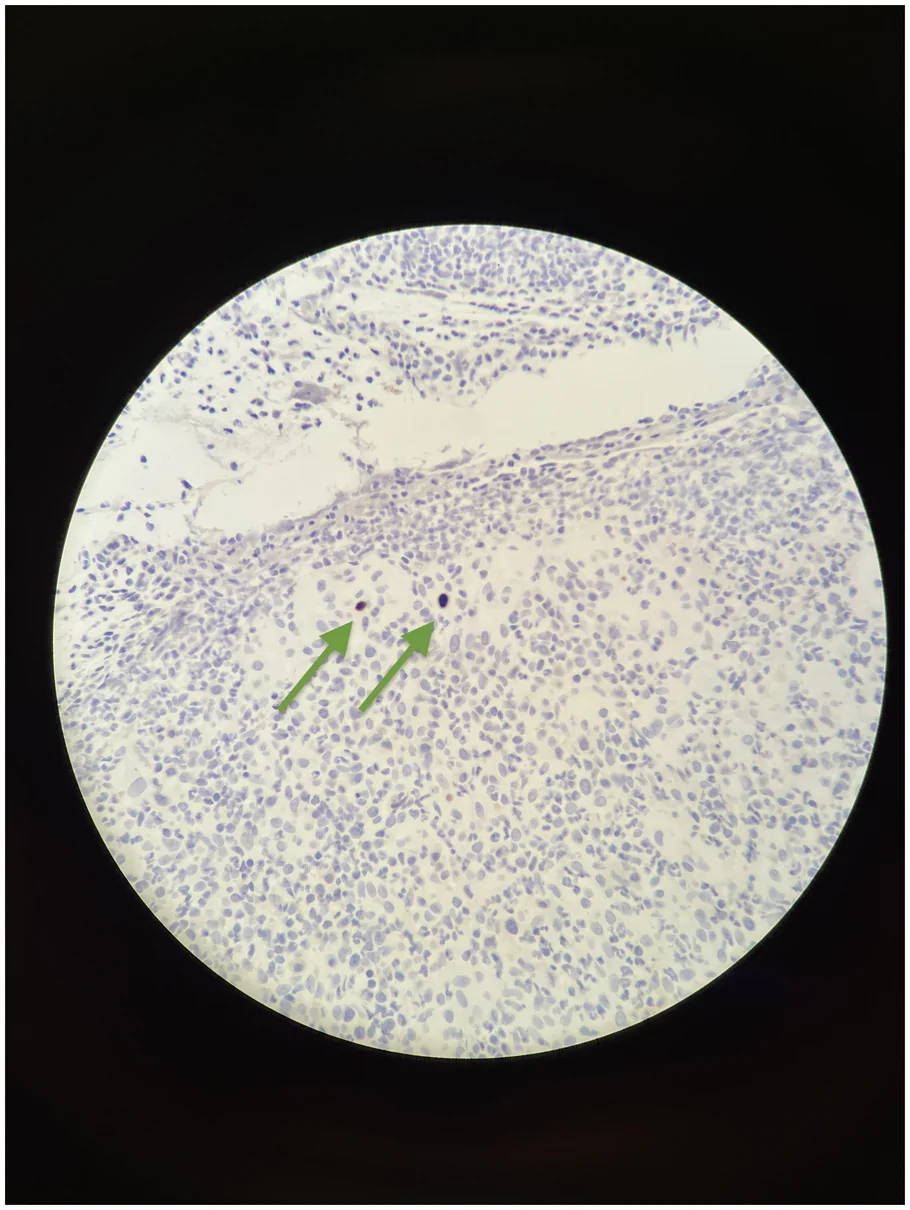
Histological image of the small bowel ulcer, showing positive CMV immunostaining (arrow).

The diagnosis was CMV small-bowel ulcer, which is a tissue-invasive CMV disease. This had caused the substantial gastrointestinal bleeding.

On day 8 of admission (before enteroscopy), a CMV pp65 assay was performed, which yielded negative results. CMV pp65 detects antigen inside leukocytes and can be falsely negative in immunosuppressed patients (10). On the same day, CMV DNA PCR assay revealed raised values at 1.90 × 10^3^ IU/mL.

### Outcome and follow-up

As of day 10 of admission, the patient had no further passage of blood, and his Hb levels improved to around 9 g/dL. He was discharged on day 18 of admission.

We performed a follow-up 4 days after discharge. We reviewed his histology report, which showed CMV small-bowel ulcer. In view of his immunocompromised status, a total of 3 weeks of oral valganciclovir 450 mg daily (renal adjusted dose) was initiated.

Blood samples were taken for CMV DNA PCR assay on the day of follow-up (4 days after discharge), which yielded negative results before the first dose of antiviral medicine. This might have been due to the reduction of immunosuppressant (stopped MMF), causing some restoration of immunity. CMV DNA PCR assay was performed again at week 1 and 3 of initiating antiviral therapy; all results came back negative.

Follow-up was continued weekly for two more weeks, with the patient displaying no passage of blood. His Hb levels were static.

A follow-up CLN was performed 1 month after the index enteroscopy. It showed erythematous areas over the ICV with some scarring. The patchy erythematous areas over the terminal ileum probably correlate with the previous ulcers.

## Discussion

### Strengths and limitations of our approach

The strength of our approach was the multi-disciplinary management, involving infectious disease physicians, surgeons, gastroenterologists, nephrologists, and the nursing and support staff. This enabled us to handle the patient in a holistic way, taking care of all the involved specialties.

One of the limitations of our approach was the time required to establish tissue diagnosis. DBE was performed on day 12 of admission, when the bleeding had already subsided. There was a delay, with small-bowel bleeding being suspected on day 5 and the DBE being performed on day 12. This delay was due to technical and instrumental issues. Unlike OGD and CLN which are widely available, DBE is a highly technically demanding, uncommon procedure, even for gastroenterologists. Our hospital, and many hospitals in our city, did not have an in-house DBE. We needed to borrow it from a medical supplier, which took a few days. In hospitals without the expertise to perform a DBE, patients may need to be transferred to a specialised centre, causing further delay.

These limitations illustrate the intrinsic difficulties in managing small-bowel bleeding. It is technically difficult to obtain tissue diagnosis. As in our case, if DBE had been performed earlier, we might have been able to stop the bleeding by endoscopic methods such as applying haemostatic clips.

### Brief discussion on CMV disease

CMV is a member of the herpesvirus (human herpesvirus 5) that has a worldwide distribution. It is usually acquired in childhood or early adulthood. CMV replicates in the nuclei of a variety of cells, such as epithelial cells, endothelial cells, fibroblasts, and leukocytes. After the initial phase, it enters into latency in myeloid cells in the bone marrow (11, 12). Sometimes in the later life, CMV replicates again, causing reactivation. An immunocompromised status is a known risk factor for CMV reactivation (13). Active replication of CMV in the body is termed CMV infection. It can be symptomatic or asymptomatic. If symptoms exist, it is termed CMV disease (14).

As mentioned earlier, CMV disease can involve every organ or system. It can involve the whole gastrointestinal tract from the mouth to the anus. Small-bowel involvement is a rare manifestation of the virus (3).

### CMV involving small bowel

The small bowel is a rare location for CMV infection. From the reported cases of CMV involving the small bowel in particular, there are several morphologies.

A diffuse swelling with erosions over the small bowel is a commonly reported morphology (4, 15, 16). If there is marked mucosal swelling, it might be visible through computed tomography (CT) scans (15). Discrete erosions and ulcers often occur in multiples and involve a long segment of the gastrointestinal tract (17). The morphology in our case—a few small discrete ulcers—is a rarely reported one (18). Small discrete ulcers, as in our case, prove diagnostically difficult. An ulcer without surrounding oedema is rarely visible in cross-sectional imaging. Even during endoscopy, small and sparse ulcers are difficult to detect.

If left untreated, ulcers might progress, resulting in complications. Bleeding is the most common complication. However, in some cases, small-bowel perforation is the presenting morphology, requiring surgical intervention (5, 19–22).

### Proposed mechanism and pathophysiology of CMV infection in our case

In our case, the bleeding resolved on day 9–10 before the antiviral course was initiated. The clinical improvement might have been due to the withdrawal of MMF. In normal individuals, CMV replication is inhibited by the T lymphocytes (23). MMF is an immunosuppressant that suppresses lymphocyte proliferation (24), possibly increasing the risk of CMV reactivation. The association between MMF and CMV infection in transplant recipients has been shown in earlier studies (25, 26). The withdrawal of MMF allowed the body to restore some cell-mediated immunity, leading to the cessation of bleeding from CMV ulcers. A reduction in immunosuppressant is therefore important in treating CMV infection (27).

Misoprostol is an FDA-approved drug for treating non-steroidal anti-inflammatory drug (NSAID)-induced gastric ulcers (28), and there is some evidence for this drug in treating aspirin or NSAID-induced small-bowel ulcers as well (29, 30). We started misoprostol on day 8 of admission. At that juncture, we suspected small-bowel bleeding, and were in the process of arranging DBE, but it was not yet available due to technical and instrumental issues while the patient was still experiencing active bleeding. Our rationale for starting misoprostol was based on two factors: First, drug-induced small-bowel bleeding remained a possibility for our patient; second, being a prostaglandin analogue, misoprostol can enhance mucosal defence and cellular repair in the gastrointestinal epithelium (28). Thus, it might be helpful for bleeding lesions in the small bowel. After a careful explanation of the off-label use of misoprostol, and the possible adverse effects, our patient agreed to it. We initiated misoprostol 200 μg four times a day on day 8 of admission.

An early follow-up was arranged 4 days after discharge. By that time, the patient’s Hb levels were static and his symptoms had already subsided. During his hospital stay, CMV DNA PCR was checked to be raised to 1.90 × 10^3^ IU/mL on day 8 of admission. On the day of follow-up (4 days after discharge), just before starting valganciclovir, CMV DNA PCR was checked to be negative already. This indicated that CMV viraemia had already subsided, possibly due to stopping MMF. However, it should be noted that CMV viraemia does not necessarily correlate with the disease activity of tissue-invasive disease. The clearing-up of viraemia does not mean that the CMV ulcers have healed. In view of the patient’s immunocompromised status, a course of antiviral medication was still indicated. The timeline of clinical course and intervention is summarised in Figure 4 (See Figure 4).

**Figure 4:**
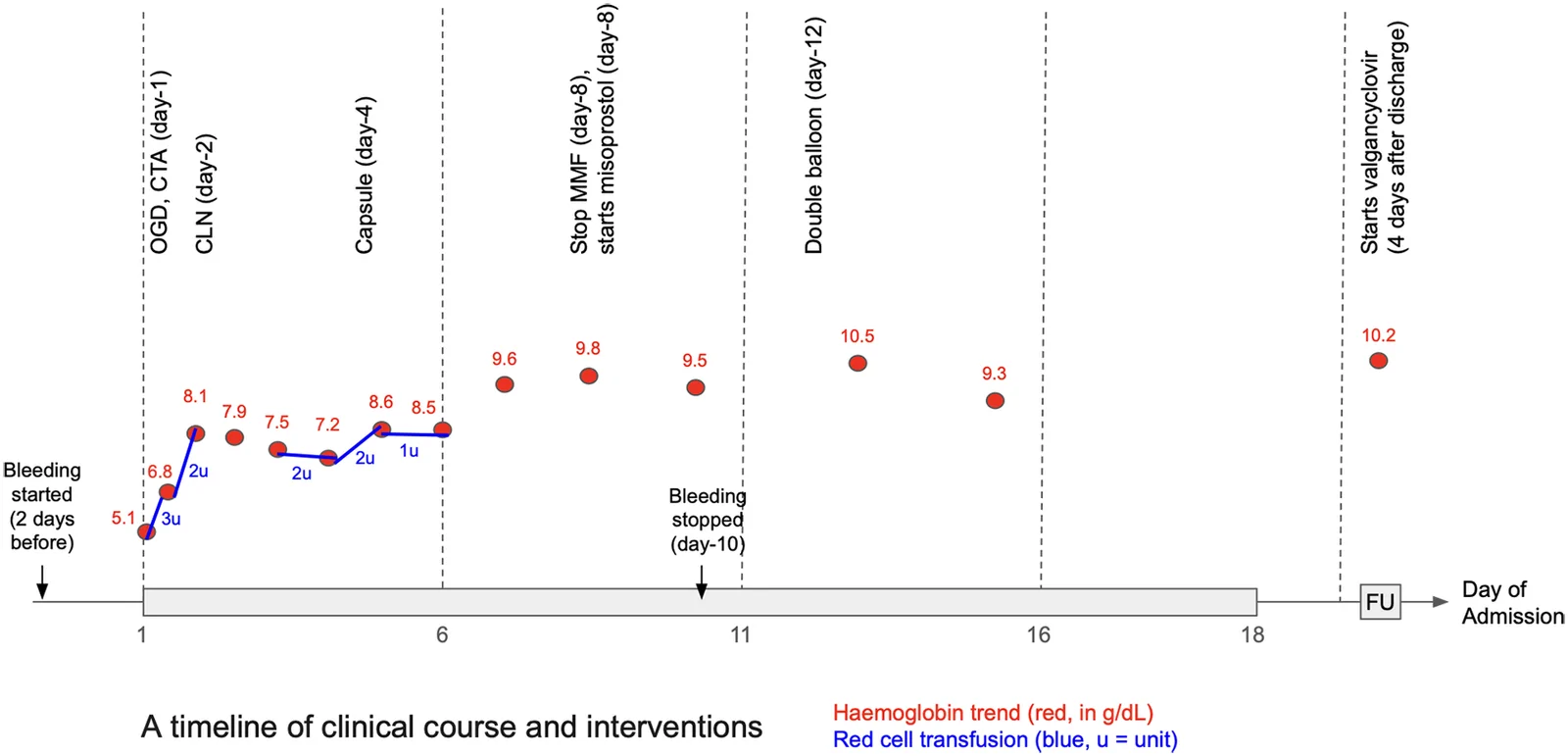
A timeline of clinical course and interventions.

### Consent

Informed consent was obtained from the patient for the publication of this case report.

## Conclusion

We reported a rare case of tissue-invasive CMV disease, that is, small-bowel ulcers causing significant gastrointestinal bleeding. Due to the technical difficulty in accessing the area, small-bowel bleeding is often not considered during diagnosis, particularly during the initial assessment of gastrointestinal bleeding. However, in immunocompromised patients, CMV can involve every part of the gastrointestinal tract. The treatment involves both adjustment of immunosuppressants and use of antiviral medication.

## Data Availability

The raw data supporting the conclusions of this article will be made available by the authors without undue reservation.
